# MiR-223 targeting MAFB suppresses proliferation and migration of nasopharyngeal carcinoma cells

**DOI:** 10.1186/s12885-015-1464-x

**Published:** 2015-06-09

**Authors:** Wanyong Yang, Xi Lan, Dongmin Li, Tao Li, Shemin Lu

**Affiliations:** 1 Department of Biochemistry and Molecular Biology, School of Medical Basic Sciences, Xi’an Jiaotong University Health Science Center, Xi’an, Shaanxi 710061 P. R. China; 2 Dongguan Guanghua Hospital, Dongguan, Guangdong 523416 P. R. China; 3 Guangdong Provincial Key Laboratory of Medical Molecular Diagnostics, Guangdong Medical College, Dongguan, Guangdong 523808 P. R. China

**Keywords:** MiR-223, Nasopharyngeal carcinoma, MAFB, Proliferation, Migration

## Abstract

**Background:**

Mounting evidence suggests that miRNAs have major functions in tumor pathogenesis, and this study aimed to identify the candidate miRNA and investigate its role in nasopharyngeal carcinoma (NPC).

**Methods:**

MiRNA and mRNA expressions were screened by microarray assays. The cell proliferation, colony formation and migration ability were measured by MTT, soft agar and wound healing assays, respectively. The tumor growth suppression was evaluated by xenografting in nude mice. The plasma miR-223 levels in NPC patients were detected by TaqMan analysis. Real-time quantitative PCR and Western blotting were used to confirm miR-223 and MAFB expression levels. The targeting relationship between miR-223 and MAFB was verified using dual luciferase reporter assay.

**Results:**

The miR-223 expression was decreased in CNE-1, CNE-2 cells as compared with NP69 cells, an immortalized human nasopharyngeal epithelial cell line, and its level also reduced in NPC patients’ plasma as compared with healthy controls. Exogenous expression of miR-223 in CNE-2 cells could inhibit cell proliferation both in vitro and in vivo. Extrogenous miR-223 in CNE-2 cells would decrease the ability of colony formation and migration. MAFB, a transcription factor of Maf family members, was identified as a target gene of miR-223. We found that migration and invasion abilities were inhibited by MAFB silencing.

**Conclusions:**

MiR-223 negatively regulates the growth and migration of NPC cells via reducing MAFB expression, and this finding provides a novel insight into understanding miR-223 regulation mechanism in nasopharyngeal carcinoma tumorigenesis.

## Background

Nasopharyngeal carcinoma (NPC) is a common malignant tumor in the people of southern China, particularly in Guangdong population [1]. Radiotherapy is a commonly used method to treat NPC and combined with chemotherapy to promote the survival rate of the patients. However, NPC cells can easily invade local tissue even metastasize to remote organs, so such relapse and metastasis result in poor prognosis for the patients [2]. Therefore it is very important to further elucidate pathogenesis of NPC for discovering new therapeutic approaches.

MicroRNAs (miRNAs) are endogenous non-coding RNAs with approachable 22 nucleotides in length and play an important role in physiological and pathological conditions through cleaving or transcript suppressing target mRNAs [3]. Accumulating evidence demonstrates that miRNAs are associated with cancer occurrence. And determination of miRNA levels has been proposed as a biomarker for diagnosis and prognosis of various cancers [4, 5]. Here, we identified miR-223 as down-regulated in undifferentiated nasopharyngeal carcinoma cell line CNE-2, compared with immortalized nasopharyngeal epithelial cell line NP69. MiR-223 was firstly reported to be involved in the regulation of human granulopoiesis [6, 7]. Then it was found to be a potential biomarker for recurrent ovarian cancer [8]. In Hela cells, overexpression of MiR-223 suppresses cell proliferation by targeting IGF-1R [9]. It seems contradictory that miR-223 can suppress tumor invasion and metastasis through targeting Artemin [10], but may promote tumor by inhibiting the expression of EPB41L3, a tumor suppressor in human NPC [10]. These findings suggest that miR-223 is associated with migration and invasion of malignant tumor. However, to our knowledge, the role of miR-223 in nasopharyngeal carcinogenesis remains undefined.

The present study was performed to find the potential miRNAs in NPC, and verify the role of target miRNA in invasion and metastasis of the cells. Our results illuminate the role of miR-223 in NPC development and provide valuable information for clinical implications.

## Materials and methods

### Cell culture

Highly and poorly differentiated human NPC cell lines named as CNE-1 and 2 were established and kindly provided by Prof. Yi Zeng from the Institute of Virology, China Institute of Preventive Medical Science, China [11]. The immortalized human nasopharyngeal epithelial cell line named as NP69 was kindly provided by Prof. Kaitai Yao from Southern Medical University, Guangzhou, China. CNE-1 and CNE-2 cells were cultured in the DMEM medium containing 10 % fetal bovine serum (FBS), and NP69 cells were cultured in Keratinocyte-SFM (serum-free medium). Both mediums were contained 100 units/ml penicillin G and 100 μg/ml streptomycin (Invitrogen). The transfection was conducted with Lipofectamine™2000 reagent (Invitrogen).

### Microarray analysis

For microarray assay of miRNAs, double-stranded cDNAs were synthesized with the 2 μg total RNA, and biotin-tagged cRNAs were obtained by the use of the MessageAmp™ II aRNA Amplification Kit (Ambion). The biotin-tagged cRNAs were fragmented into mixed strands in accordance with the Affymetrix’s protocols. The fragmented cRNAs were hybridized with TaqMan® Human MicroRNA Array Set v3.0 containing 754 transcripts. Hybridization was performed at 45 °C with rotation for 16 h (Affymetrix GeneChip Hybridization Oven 640). The GeneChip arrays were washed and then stained (streptavidin-phycoerythrin) on an Affymetrix Fluidics Station 450 followed by scanning on a GeneChip Scanner 3000.

### MiRNA transfection and real-time quantitative PCR

MiR-223 mimic (Cat^#^ miR10004570), miR-223 negative control (Cat^#^ miR01201), miR-223 (Ca^t#^ miRQ0004570) and U6 (Cat^#^ MQP-0201) real-time PCR primers RNA oligonucleotides were obtained from RiboBio (http://www.ribobio.com Guangzhou, China). miRNAs were transfected to CNE-2 cells by using Lipofectamine®2000, with a final concentration at 50 nM. The expression of miR-223 was measured by real-time quantitative PCR (RT-qPCR) by using One Step SYBR® PrimeScript® RT-PCR Kit II (Takara) and following the manufacturer’s protocol on ABI 7500 HT real-time PCR detection system as normalized to the housekeeping gene U6. The information about primer sequences is depicted in Table 1.

**Table 1:** Genes and primers for RT-qPCR

Gene	Primer name	Sequence (from 5’ to 3’)
*U6*	Reverse transcription	CGCTTCACGAATTTGCGTGTCAT
	Sense	GCTTCGGCAGCACATATACTAAAA
	Anti sense	GCTTCGGCAGCACATATACTAAAAT
*miR-223*	Reverse transcription	GTCGTATCCAGTGCGTGTCGTGGAGTCGGCAATTGCACTGGATACGACTGGGGT
	Sense	GGGTGTCAGTTTGTCAAAT
	Anti sense	TGCGTGTCGTGGAGTC
*GAPDH*	Sense	GCACCGTCAAGGCTGAGAAC
	Anti sense	TGGTGAAGACGCCAGTGGA
*MAFB*	Sense	TTGTAACCAGAATCACCCTGAGGTC
	Anti sense	CCAGGGTCAGGGATGGCTAA

### Cell proliferation analysis

CNE-2 cells with a number of 5 × 10^3^/well were seeded into 96-well plates, incubated for 12 h, and then transfection was performed. At 24 h after transfection, each well was treated with 10 μL MTT solution (10 mg/ml in PBS) and incubated sequentially at 37 °C. After the incubation for 4 h, 100 μL of DMSO was added to dissolve the crystals. Then the absorbance of each well in culture plate was measured at 570 nm and 630 nm after oscillated for 10 min at room temperature.

### Colony formation assay

Two experiments were employed for measuring colony formation ability. For plate colony formation, at 24 h after transfection, 600 cells were plated for 10 days. The colonies were photographed and counted. For soft agar analysis, 1 × 10^4^ cells were plated in triplicates in 6 cm diameter plates with 0.6 % base agar and 0.3 % top agar and incubated 21 days. The colonies were count in 10 randomly chosen microscope fields.

### Wound healing assay

The cells (2 × 10^5^) were seeded into a 6-well plate. Then the cells were scratched using a 100 μl tip when the cells formed a confluent monolayer. The closure of scratch was detected under the microscope at 0, 24, 48, 72 h time point respectively after incubation.

### Transwell migration and invasion assay

The ability of cells to migrate through filters was measured using Polycarbonate Membrane Transwell Inserts (Corning). At 24 h after transfection, cells were trypsinised. Cell culture inserts with a polycarbonate membrane (8 μm pore size) were used. The bottom chamber contained medium (0.5 ml) supplemented with 10 % FBS, whereas the transfected cells were seeded into the upper chamber and incubated for 36 h. The remaining cells on the upper surface were mechanically removed. Then the membranes were washed and stained using 0.1 % crystal violet. The cell migration ability was measured by the cell numbers that migrated to the lower side of the filter. Experiments were repeated 3 times.

The invasion ability of cells was determined by a Matrigel invasion chamber assay. This assay was conducted by the use of 6.5 mm and 8 μm pore size Transwell chambers (Corning). Cells transfected with miRNA mimic or control one were cultured in serum-free medium for 12 h (1 × 10^5^ cells per Transwell) and then the cells were migrated into a medium containing 10 % FBS for 24 h. Cells that migrated to the underside of the filter were fixed in methanol and stained using 0.1 % crystal violet. Whole filters were manually counted under the inverted microscope.

### In vivo antitumor assay

Seven nude athymic mice (male, SPF grade, 6–8 weeks of age) were purchased from Animal Experimental Center of Guangdong Medical College. CNE-2 cells transfected with miR-223 mimic and control microRNA (2 × 10^6^) or MAFB specific siRNA and negative control siRNA in 100 μl no serum medium were injected respectively into particular side of each mouse. Tumor size was measured every three day and tumor volume was calculated as V = ab^2^/2, where a is length and b is width of tumor. At day 23 after cell injection, the animals were sacrificed and the tumors were frozen quickly for following assays. Tissue sections were fixed in 4 % paraformaldehyde and embedded in paraffin. Three micrometer tissue sections were prepared and stained with hematoxylin-eosin. All of the animal experiments were approved by the Experimental Animal Care and Use Committee in Guangdong Medical College.

### Patient plasma collection

Human plasma samples from NPC patients and healthy donors were derived from the Affiliated Hospital of Guangdong Medical College and Dongguan area in China, with the informed consent under institutional review board-approved protocols. Blood was collected in EDTA tubes and processed for isolation of plasma within 2 h of collection. Blood was centrifuged at 2000 rpm at room temperature for 10 min. Then the upper layer plasma was transferred to RNase/DNase-free 1.5 ml conical tubes and stored at −80 °C.

Total RNA was enriched from all plasma samples using the TRIzol LS (Invitrogen). To normalize the different RNA samples, 10 μl of synthetic cel-miR-39 (1.6 × 10^8^ copies/μl) was added to each sample during the RNA isolation. The reverse transcription was conducted using the TaqMan miRNA Reverse Transcription Kit (Life Technologies), according to the manufacturer’s protocol. RT-qPCR was carried out as described above. Data were analyzed under the SDS Relative Quantification Software version 1.4 (Applied BioSystems).

### Bioinformatics and dual luciferase reporter gene assay

Two widely advocated online bioinformatic softwares, Target Scan [12] and microRNA.org [13], were used to predict the interaction probability between miR-223 and *MAFB*, including binding sites for miR-223 in MAFB and the targeting efficiency. The predictive outcome was used for further investigation.

A 249-bp-long MAFB 3’UTR fragment was cloned into downstream region of the luciferase gene within the pGL3 vector (Promega), namely the wild-type 3’UTR (WT-UTR). The mutated MAFB 3’UTR containing mutations of the miR-223 binding sit was obtained using GeneTailor Site-Directed Mutagenesis System (Invitrogen), namely mutated 3’UTR (mt-UTR). For dual luciferase reporter assays, WT-UTR or mt-UTR recombinant vector (0.5 μg per well) was cotransfected with the normalizer vector pRL-CMV (0.5 μg per well per well) coding for Renilla luciferase (Promega) using Lipofectamin 2000™ (Invitrogen). The CNE-2 cells were cultured in 24 wells plates with the density of 5 × 10^5^ per well. Dual-Luciferase® Reporter 1000 Assay System (Promega) was used to detect the luciferase activity at 36 h after transfection.

### cDNA microarray and confirmation

cDNA microarray analysis was performed on Human Genome U133 Plus 2.0 gene chips by Capital Bio Corporation (Beijing, China). The microarray results were confirmed by RT-qPCR, with housekeeping gene GAPDH as a normalizer.

### MAFB specific siRNA transfection

MAFB siRNA and negative control were synthesized by RiboBio (Guangzhou, China). The transfection was performed using Lipofectamin 2000™ (Invitrogen). Then the cells were harvested 24 h after transfection, and the MAFB expression level was measured by RT-qPCR as described above.

### Western blotting

The total cell lysates were harvested and the total protein was separated on SDS-PAGE gel by Bio-rad system. The anti-MAFB and anti-GAPDH antibodies were used as the primary antibody (Abcam), with GAPDH as a normalizer. The signal intensity was detected with the enhanced chemiluminescence substrate kit (Thermo).

### Statistical analysis

Data were expressed as means ± standard deviation (SD), and the the analysis between groups was performed by Student’s *t*-test unless otherwise noted. Difference with a *P*-value less than 0.05 was considered significant.

## Results

### MiR-223 expression is significantly lower in CNE samples than control samples

**Fig. 1:**
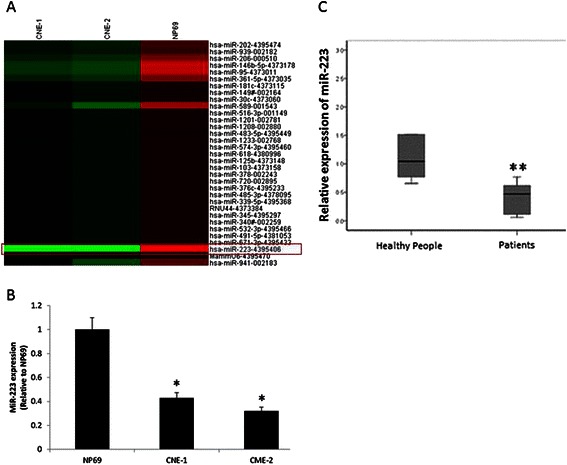
MiR-223 is down-regulated in human NPC cell lines and patients. **a**. The miRNA microarray analysis revealed that there were 33 differentially expressed miRNAs between CNE-1, CNE-2 and NP69 (changed more than 5 folds). **b**. RT-qPCR confirmed that the miR-223 expression was significantly lower in CNE-1 and CNE-2 than that in NP69 (*P < 0.05). The miR-223 expression was analyzed with U6 as a normalizer. The data were showed as means ± SD from three independent experiments. **c**. The miR-223 expression was significantly decreased in NPC patients’ plasma. MiRNA abundance was normalized to cell-miR-39. The data were showed as means ± SD (n = 10)

MiRNA expression profiling was conducted with a miRNA microarray using RNAs isolated from human nasopharyngeal carcinoma cell line CNE-1, CNE-2 and human immortalized nasopharyngeal epithelial cell line NP69. A total of 754 miRNAs were detected, in which 25 were increased and 8 were decreased more than 5 folds in NPC cells, compared with NP69 cells (Fig. 1a). MiR-223, which was recently identified to be related with migration and invasion of malignant tumors, was lower expressed in NPC cell lines CNE-1 and CNE-2. We employed RT-qPCR to validate the microarray results and a consistent result was observed (P < 0.05; Fig. 1b). The expression level of miR-223 was also down-regulated in another two NPC cell lines HONE-1 and SUNE-1 (data no shown). These results encouraged us to check the expression level of miR-223 in clinical specimens. We found that the average miR-223 level was significantly lower in the plasma of 10 NPC patients than that from 10 normal healthy subjects.

### Exogenous miR-223 inhibits cell proliferation and colony formation in vitro

**Fig. 2:**
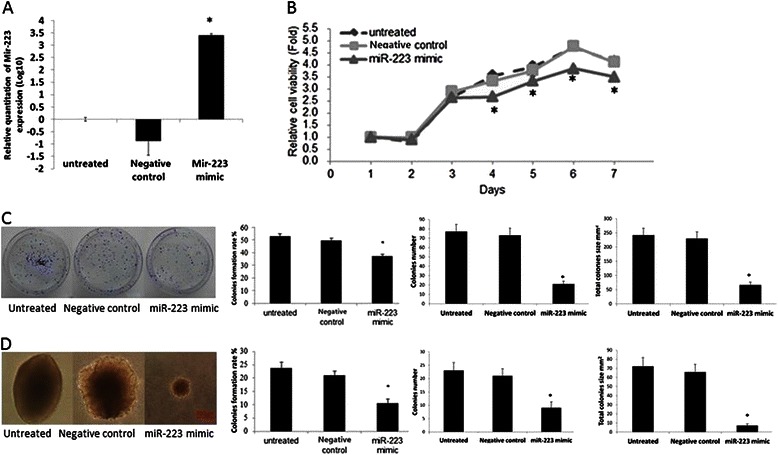
Exogenous miR-223 suppresses cell proliferation and colony formation in vitro. **a**. RT-qPCR result confirmed that the miR-223 expression was significantly higher as compared with untreated and control group after exogenous miR-223 transfection in CNE-2 (*P < 0.05). **b**. MTT cell viability was assayed one time a day from day 1 to day 7 after the transfection of either miR-223 mimic or the negative control in CNE-2 cells. **c**. After the transfection with miR-223 mimic or the negative control in CNE-2 cells and the 10 days incubation on plates, the colony formation assay was performed. The colony formation rate, clone number and total clone size were calculated respectively. **d**. After the transfection with miR-223 mimics or the negative control in CNE-2 cells and the 21 days incubation on soft agar, the colony formation assay was performed. Colonies were counted in 10 randomly chosen microscope fields and the colony formation rate, clone number and total clone size was calculated. We performed at least three independent experiments. The data were showed as means ± SD. * indicates P < 0.05 compared with control group

In vitro, the cells transfected with miR-223 mimics showed a higher miR-223 expression level (Fig. 2a). The potential role of miR-223 mimic in cell proliferation and colony formation was observed. As a result, the growth rate was significantly decreased in CNE-2 cells due to exogenous miR-223 (Fig. 2b). This observation suggested that miR-223 may inhibit the proliferation of NPC cells. This notion was further supported by the colony formation assay. As shown in Fig. 2c and d, both plate and soft agar colony formation abilities were suppressed by exogenous miR-223 in CNE-2 cells.

### MiR-223 inhibits NPC cell migration and invasion

**Fig. 3:**
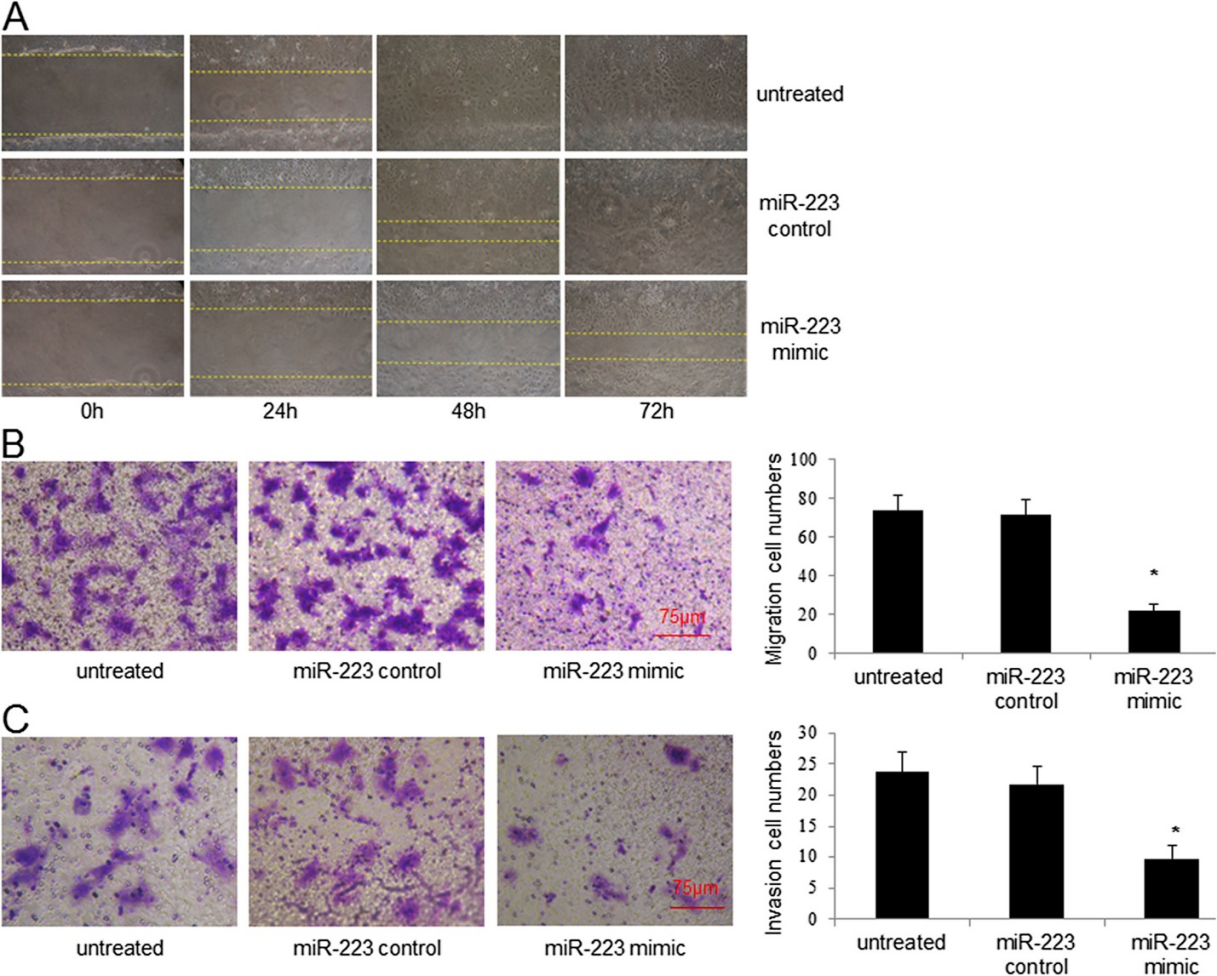
Exogenous miR-223 suppresses cell migration and invasion in vitro. **a**. The function of exogenous miR-223 on CNE-2 cells migration. CNE-2 cells were cultured in 6-well plates. After the wound was made, images were captured at 0 h, 24 h, 48 h and 72 h time point respectively. **b**. The effect of exogenous miR-223 on CNE-2 migration through Membrane Transwell assay. **c**. The effect of miR-223 on CNE-2 invasion by Matrigel invasion assay. The experiments were conducted at least 3 times. The data were showed as means ± SD. * indicates P < 0.05 compared with control group

The proliferation inhibition of miR-223 in CNE-2 cells prompted us to examine the effect of miR-223 on the migration and invasion of NPC cells. By wound healing assay, we found that exogenous miR-223 inhibited CNE-2 cell migration (Fig. 3a). Consistently, the results of in vitro transwell migration and Matrigel invasion assay showed that exogenous miR-223 inhibited CNE-2 cells migration and invasion (Fig. 3b and c). These results demonstrated that miR-223 inhibits the migration and invasion of NPC cells in vitro.

### MiR-223 suppresses tumor growth in vivo

**Fig. 4:**
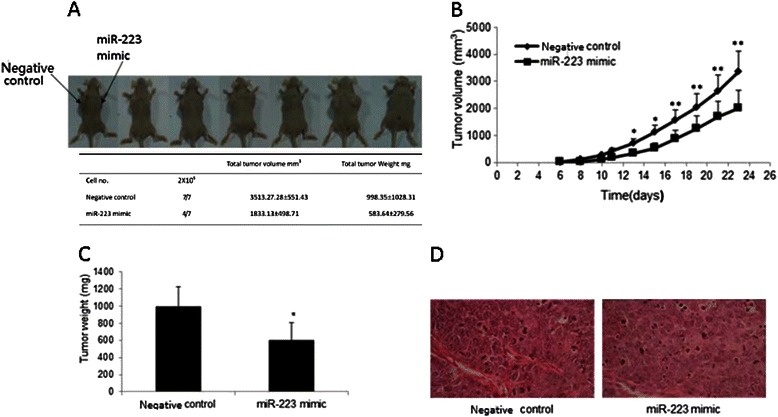
Exogenous miR-223 attenuates nasopharyngeal tumor growth in mouse xenograft models. **a**. The appearance of xenograft subcutaneous NPC in miR-223 mimics transfection and control groups. **b**. Growth curves of subcutaneous NPC in nude mice with miR-223 mimics transfection revealed that tumor growth significantly slowed down compared with control group. **c**. Total tumor weight of miR-223 mimic transfection and control groups. **d**. HE staining result of the tumor formed in nude mice using, all of them were typical nasopharyngeal squamous cell carcinoma. The data were showed as means ± SD (n = 7). * and ** indicate P < 0.05 and P < 0.01 as compared with control group

As exogenous miR-223 suppresses cell proliferation of NPC cells in vitro, we examined the effect of miR-223 in vivo. After injected with CNE-2 cells that were transfected with miR-223 mimic or control microRNA respectively, we found that tumors grew at a slower rate and had smaller sizes (Fig. 4a-b). The total tumor size for negative control and miR-223 mimic transfected groups was 995.37 ± 674.63 and 598.38 ± 613.23 mm^3^ respectively (Fig. 4c). Morphological similarity of the xenograft to the human NPC was evident on paraffin-embedded sections stained with hematoxylin-eosin (Fig. 4d). These results indicated that exogenous miR-223 suppresses tumor growth in vivo.

### MAFB is identified as a direct target of miR-223

**Fig. 5:**
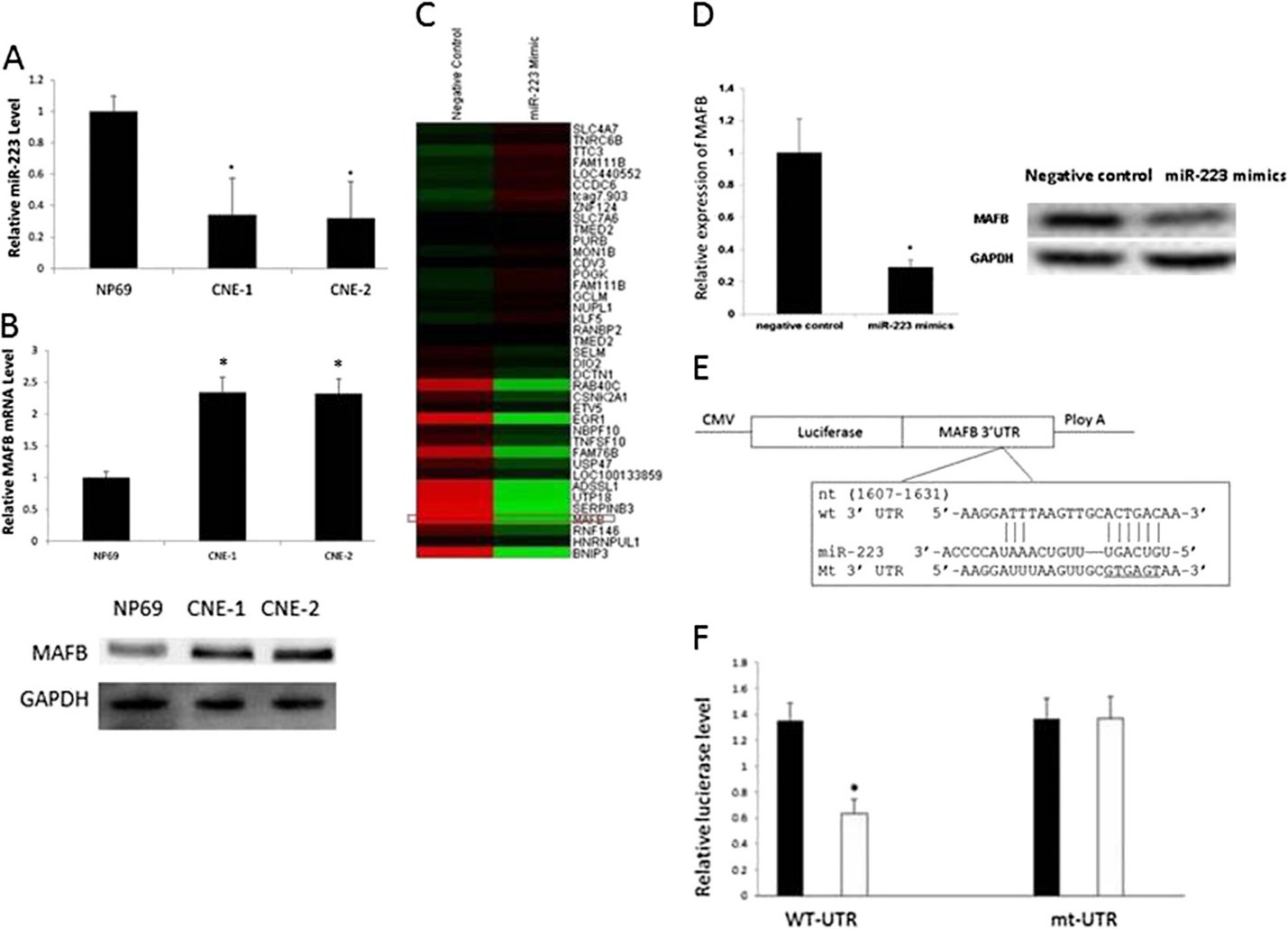
MAFB is a direct target gene of miR-223. **a**. The RT-qPCR confirmed that the miR-223 expression was significantly lower in CNE-1 and CNE-2 than that in NP69 (*P < 0.05). **b**. MAFB gene expression level in NP69 and NPC cell line CNE-1 and CNE-2 was measured by RT-qPCR and western blot. **c**. The cDNA microarray analysis revealed that there were 39 differentially expressed genes between the CNE-2 transfected with miR-223 mimics and the CNE-2 transfected with negative control. **d**. RT-qPCR confirmed that the gene expression of MAFB was inhibited by miR-223 overexpression. The inhibition of MAFB gene expression on protein level through miR-223 mimic administration was confirmed by Western blotting. **e**. Schematic diagram of MAFB 3’ UTR-containing reporter gene constructs. **f**. Dual luciferase reporter assay in CNE-2 co-transfected with WT-UTR or mt-UTR recombinant vector (0.5 μg) and miR-223 (50 nM) as indicated. The experiment was performed at least 3 times. The data were showed as means ± SD. * indicates P < 0.05 compared with control group

The miR-223 expression level in CNE-1, CNE-2 and NP69 cell lines was measured by RT-qPCR. Compared with NP69, miR-223 expression was significantly lower in NPC cell line CNE-1 and CNE-2 (Fig. 5a) that was consistent with the miRNA microarray results (Fig. 1a). The MAFB gene expression in CNE-1, CNE-2 and NP69 was also checked through RT-qPCR and Western blotting. As shown in Fig. 5b, MAFB gene expression was significantly higher in CNE-1, CNE-2 cells compared with NP69. To investigate the mechanism of miR-223 inhibition NPC cells, we performed a cDNA expression microarray analysis. After 24 h transfection of miR-223 mimic and negative control in CNE-2, total RNA was isolated and hybridized to an Affymetrix GeneChip Human Genome U133 Plus 2.0 Array. We found that 39 transcripts were differentially expressed between two groups (Fig. 5c). The microarray results for miR-223 down-regulated MAFB gene expression were verified through RT-qPCR and Western blotting. We obtained the consistent result that miR-223 suppressed MAFB gene expression in CNE-2 cells (Fig. 5d). Therefore, we focused on MAFB and observed its role on CNE-2 cell biology function. Combined bioinformatics results (Fig. 5e) with the dual luciferase reporter assay (Fig. 5f), we identified MAFB as one of miR-223 target genes for further investigation.

### MAFB siRNA inhibits CNE-2 cells growth and migration

**Fig. 6:**
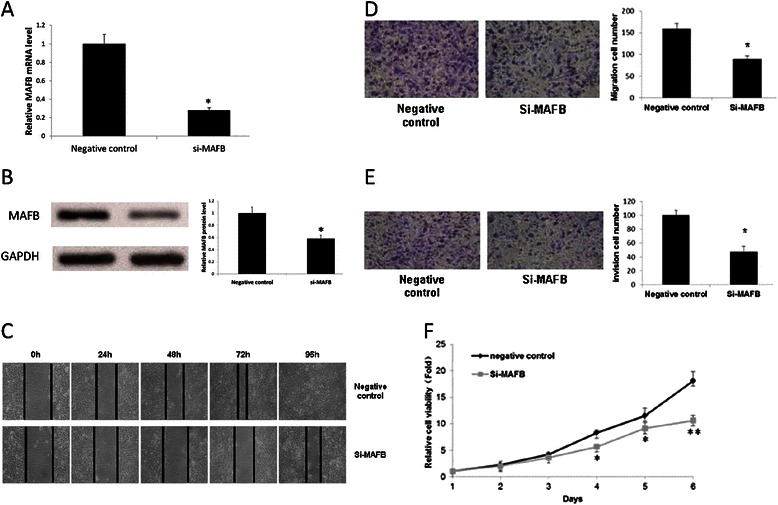
Inhibition of MAFB gene expression suppresses cell migration, invasion and proliferation in vitro. **a**. RT-qPCR confirmed that the gene expression of MAFB was inhibited by MAFB specific siRNA. **b**. Western blotting shown the inhibition of MAFB gene expression on protein level through MAFB specific siRNA administration. **c**. The effect of MAFB-siRNA on CNE-2 migration. CNE-2 cells were cultured in 6-well plates. Images were captured at 0 h, 24 h, 48 h and 72 h respectively, after the wound was made. **d**. Effect of MAFB specific siRNA on CNE-2 cells migration through Membrane Transwell assay. **e**. Effect of MAFB specific siRNA on CNE-2 cell invasion by Matrigel invasion assay. **f**. MTT cell viability was assayed once per day from day 1 to day 6 after the transfection of MAFB specific siRNA or the negative control in CNE-2 cells. The experiments were performed at least 3 times. The data were showed as means ± SD. * indicates P < 0.05 compared with control group

The inhibition activity for MAFB specific siRNA on CNE-2 MAFB gene expression was confirmed by RT-qPCR and Western blotting. MAFB specific siRNA could attenuate CNE-2 cells MAFB gene expression for 28 % in mRNA level and 58 % in protein level respectively (Fig. 6a and b). By wound healing assay, we found that MAFB specific siRNA could inhibit CNE-2 cell migration (Fig. 6c). The inhibition activity of MAFB specific siRNA in CNE-2 cells proliferation and colony formation was observed. Consistently, results from in vitro transwell migration and Matrigel invasion assay showed that MAFB specific siRNA inhibited CNE-2 cell migration and invasion (Fig. 6d and e). As a result, down-regulation of MAFB could significantly decrease the growth rate of CNE-2 cells (Fig. 6f). These results indicated that miR-223 inhibits the growth and migration of NPC cells through reducing MAFB gene expression.

### MAFB siRNA inhibits the growth of NPC xenograft tumors

**Fig. 7:**
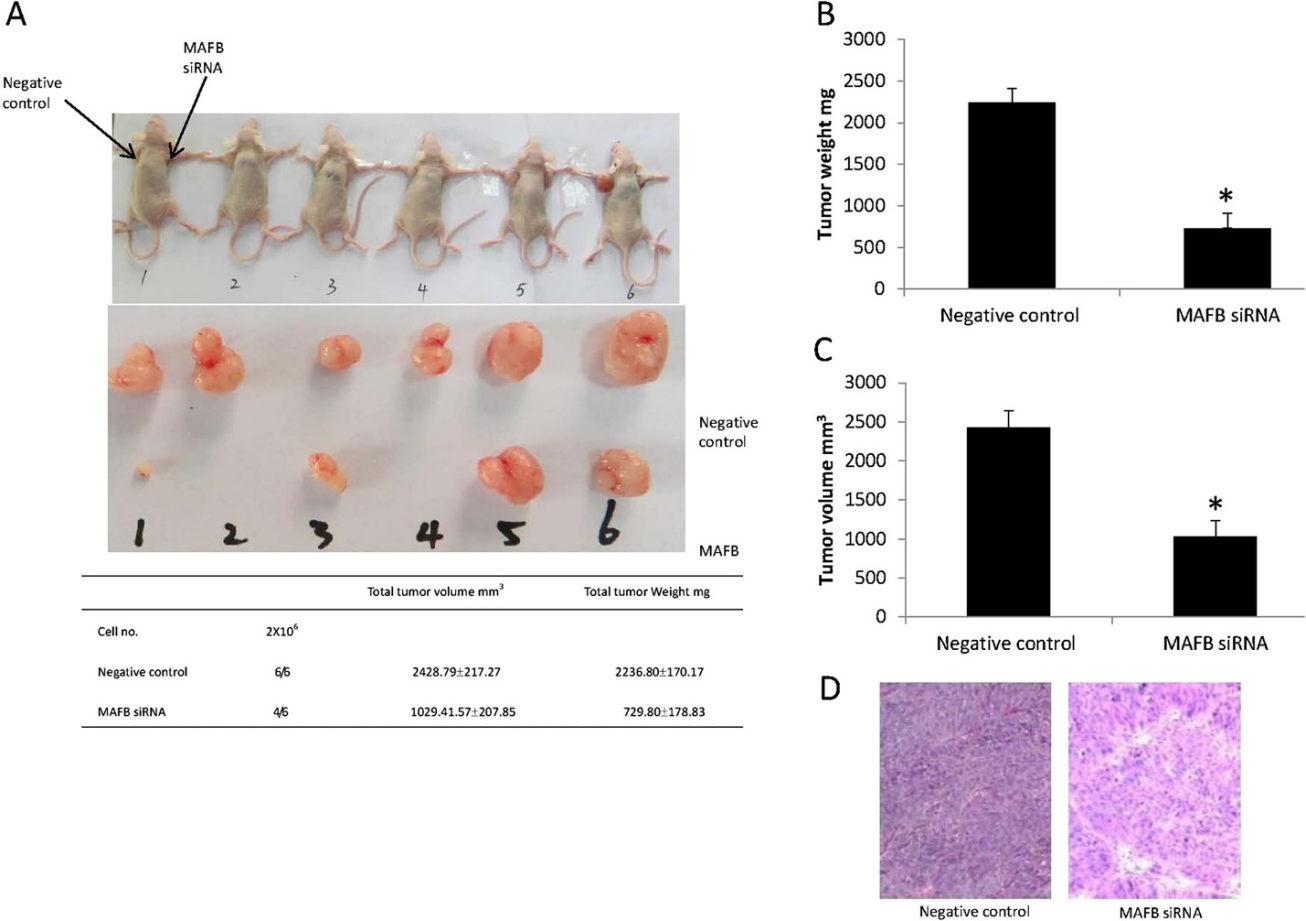
Inhibition of MAFB gene expression attenuates nasopharyngeal tumor growth in mouse xenograft models. **a**. The appearance of xenograft subcutaneous NPC in MAFB specific siRNA transfection and control groups. **b**. Total tumor weight of MAFB specific siRNA transfection and control groups. **c**. Total tumor size of MAFB specific siRNA transfection and control groups. **d**. HE staining result of the tumor formed in nude mice using, all of them were typical nasopharyngeal squamous cell carcinoma. The data were showed as means ± SD (n = 6). * indicate P < 0.05 as compared with control group

CNE-2 cells were transfected respectively with MAFB specific siRNA and negative control siRNA and then the transfected cells were injected subcutaneously into the different dorsal flank of nude mice. Compared with the negative control side, the tumors of MAFB siRNA side grew more slowly, with the smaller size. In all 6 mice for MAFB specific group, 4 mice could be seen tumors growth after 23 days while there was tumor growth in all negative control group (Fig. 7a). The total tumor weight and size was 2236.80 ± 170.17 mg and 2428.79.80 ± 217.27 mm^3^ for negative control group and 729.80 ± 178.83 mg and 1029.41 ± 207.85 mm^3^ for MAFB specific siRNA transfected group, respectively (Fig. 7b and c; * p < 0.05). Morphological similarity of the xenograft to the human NPC was evident on paraffin-embedded sections stained with hematoxylin-eosin (Fig. 7D). These results indicated that exogenous MAFB specific siRNA suppresses tumor growth in vivo.

## Discussion

To sum up, we report here that miR-223 plays a critical role in NPC carcinogenesis. The plasma miR-223 level was lower in NPC cells and NPC patients’ plasma, as compared with normal ones. To fully realize its effect, we observed that miR-223 inhibited NPC cell growth, migration and invasion both in vitro and in vivo. MAFB was identified as a miR-223 target gene. And MAFB expression was decreased accompanied with miR-223 overexpression. In addition, the consistent result was observed that the cell growth and migration were inhibited after MAFB specific siRNA transfected into NPC cells. The findings implicated that miR-223 could be a potential target for intervention and diagnosis to NPC.

NPC is a highly prevalent malignant cancer in south China and Southeast countries. It has been known that virus, diet and genetic makeups all are the risk factor for NPC development, but underlying pathogenesis of the disease is still poorly understood [14]. Oncogenes and tumor suppressing genes might form a very complicated network, and how they interact one another to lead to the cancer is not clear [15]. Few reports have indicated that miRNAs abnormally express in different cancers, and more researchers have been paying close attentions to molecular mechanisms of miRNAs in tumorigenesis [16]. In NPC, down-expressed miR-29c could upregulate mRNA expression of extracellular matrix genes [17], and ZEB2 and CTNNB1 low-expression induced by miR-200a inhibits the growth, migration and invasion of NPC cells [18]. In addition, miR-216b suppresses the proliferation and invasion via inhibiting KRAS expression [19]. Mir-218 exerts suppressing function to NPC by down-regulating survivin expression and SLIT2-BOBO1 pathway [20], and miR-26a acts the similar function for inhibiting cell growth and tumorigenesis through repression of EZH2miR [21]. Recent study reported that miR-663 targets p21 (WAF1/CIP1) to promote the proliferation and development of NPC [22]. Those results have shed light on which miRNAs are involved in NPC tumorigenesis, however, the role of miRNAs in pathogenesis has not been elucidated.

Based on miRNA microarray we found that miR-223 was down-regulated in NPC cell line CNE-1 and CNE-2, and further verified the role of miR-223 in NPC pathogenesis. Using microarray-based serum miRNA profiling and RT-qPCR method, Zeng et. al. compared serum miRNAs level between the patients with nasopharyngeal carcinoma and non-cancerous volunteers, and they found that miR-223 was decreased significantly in the serum of NPC patients [23]. Our results confirmed this by comparison the miRNAs expression between NPC and immortalized human nasopharyngeal epithelial cell line NP69. Exogenously up-regulating miR-223 could suppress cell proliferation and colony formation, increase tumor formation in nude mice (Fig. 2 and 4). The results are consistent with the discovery in hepatic carcinoma that the inhibition of miR-223 accompanies the enhancement of Stathmin1 expression [24]. In hematopoietic cells, miR-223 can negatively post-transcript regulate the expression of LMO2 and CEBP-β to reduce cell proliferation [25]. MiR-223 up-regulation in Hela cells inhibits cell proliferation by targeting IGF-1R [9]. However, in the gastric cancer development, miR-223 acts as an oncogene to promote cell invasion and migration via affecting expressions of EPB41L3 and FBXW7/hCdc4 genes [10, 26, 27]. In addition, miR-223 has been report to regulate the proliferation and invasion of human breast cancer cells for targeting Caprin-1 [28]. On the other hand, there has been another report suggesting that miR-223 functions as a potent tumor suppressor of the Lewis lung carcinoma cell line by targeting insulin-like growth factor-1 [29]. MicroRNA 223 is up-regulated in the multistep progression of Barrett’s esophagus and modulates sensitivity to chemotherapy by targeting PARP1 [30], it also modulates multidrug resistance via downregulation of ABCB1 in hepatocellular carcinoma cells [31]. In our study, we demonstrated that exogenous miR-223 inhibits CNE-2 cell migration and invasion (Fig. 3). The discrepant results of miR-223 role seemed to indicate that miR-223 regulates different gene expression to play different role in various cancers.

In the normal cells, miRNAs maintain the homeostasis of various life processes including proliferation, differentiation and apoptosis. During the tumorigenesis, deregulation of miRNAs can lead to various consequences since various miRNAs bind with many mRNAs to regulate gene expression [32]. Biological approaches are commonly used to verify the relationship between miRNAs and tumor phenotypes or to validate the possibility as a drug target [33]. MiR-122 is significantly higher in the serum of hepatocarcinoma patients, suggesting that miR-122 is likely as a novel biomarker for screening the patients [34]. A plasma miRNA panel is discovered for early diagnosis of hepatocarcinoma, and used in clinic to significantly reduce the frequency that optimal treatment window is missing [34]. Osteosarcoma development is associated with gene expression regulated by MiRNAs [4]. MiR-223/Ect2/p21 signaling regulates osteosarcoma cell cycle progression and proliferation [35]. A study also reported that miR-421 can be used as a biomarker to detect circulated tumor cells of gastric cancer patients [5]. In addition, many miRNAs have been discovered as a potential target for diagnosis and treatment of human diseases. In the serum from non-small lung cancer patients, miR-146b, miR-221, let-7a, miR-155, miR-17-5p, miR-27a and miR-106a are significantly reduced but miR-29c increased [36]. The above-mentioned reports with our study indicate that miR-223 could be a serum biomarker of NPC patients to give early diagnosis for optimal treatment.

MAFB, a member of Maf protein family, is a transcription factor with basic-leucine zipper structure and plays an important role in early tissue specification and terminal differentiation [37]. In multiple myeloma, MAFB can make hematopoietic stem/progenitor cells reprogramed into malignant plasma cells [38]. In addition, MAFB is expected to be highly sensitive and very specific biomarker for poor prognosis of multiple myeloma patients [39]. Further studies need to illustrate the role of MAFB in NPC carcinogenesis.

## Conclusion

In this study, we found the reduction of miR-223 expression both in NPC cells and in NPC patients’ plasma. After miR-223 mimic and negative control microRNA transfected, we found that 39 transcripts were differentially expressed between two groups. Those transcripts have not been reported as targets for miR-223. We also used Target Scan and microRNA.org software to predict the potential targets for miR-223 and combined the results to select MAFB for further investigation. MiR-223-mimic administration could lead to the suppression of MAFB expression both in RNA and protein levels. In addition, both of miR-223-mimic administration and reduction of MAFB expression all lead to the inhibition of cell growth and migration. These findings demonstrated that miR-223 regulates the NPC development by targeting MAFB expression, and implicates miR-223 as a candidate target for NPC diagnosis and treatment. As miR-223 maybe also targets other genes in nasopharyngeal carcinoma, further analyses are needed to elucidate the full spectrum of miR-223 functions.

## References

[1] Jia WH, Luo XY, Feng BJ, Ruan HL, Bei JX, Liu WS (2010). Traditional Cantonese diet and nasopharyngeal carcinoma risk: a large-scale case–control study in Guangdong, China. BMC Cancer.

[2] Razak AR, Siu LL, Liu FF, Ito E, O’Sullivan B, Chan K (2010). Nasopharyngeal carcinoma: the next challenges. Eur J Cancer.

[3] Bartel DP (2004). MicroRNAs: genomics, biogenesis, mechanism, and function. Cell.

[4] Jones KB, Salah Z, Del Mare S, Galasso M, Gaudio E, Nuovo GJ (2012). miRNA Signatures Associate with Pathogenesis and Progression of Osteosarcoma. Cancer Res.

[5] Zhou H, Xiao B, Zhou F, Deng H, Zhang X, Lou Y (2012). MiR-421 is a functional marker of circulating tumor cells in gastric cancer patients. Biomarkers.

[6] Fazi F, Rosa A, Fatica A, Gelmetti V, De Marchis ML, Nervi C (2005). A minicircuitry comprised of microRNA-223 and transcription factors NFI-A and C/EBPalpha regulates human granulopoiesis. Cell.

[7] Johnnidis JB, Harris MH, Wheeler RT, Stehling-Sun S, Lam MH, Kirak O (2008). Regulation of progenitor cell proliferation and granulocyte function by microRNA-223. Nature.

[8] Laios A, O’Toole S, Flavin R, Martin C, Kelly L, Ring M (2008). Potential role of miR-9 and miR-223 in recurrent ovarian cancer. Mol Cancer.

[9] Jia CY, Li HH, Zhu XC, Dong YW, Fu D, Zhao QL (2011). MiR-223 suppresses cell proliferation by targeting IGF-1R. PLoS One.

[10] Li T, Chen JX, Fu XP, Yang S, Zhang Z, Chen Kh H (2011). microRNA expression profiling of nasopharyngeal carcinoma. Oncol Rep.

[11] Sizhong Z, Xiukung G, Yi Z (1983). Cytogenetic studies on an epithelial cell line derived from poorly differentiated nasopharyngeal carcinoma. Int J Cancer.

[12] Lewis BP, Burge CB, Bartel DP (2005). Conserved seed pairing, often flanked by adenosines, indicates that thousands of human genes are microRNA targets. Cell.

[13] Betel D, Wilson M, Gabow A, Marks DS, Sander C (2008). The microRNA.org resource: targets and expression. Nucleic Acids Res.

[14] Yoshizaki T, Ito M, Murono S, Wakisaka N, Kondo S, Endo K (2012). Current understanding and management of nasopharyngeal carcinoma. Auris Nasus Larynx.

[15] Luo Z, Zhang L, Li Z, Li X, Li G, Yu H (2012). An in silico analysis of dynamic changes in microRNA expression profiles in stepwise development of nasopharyngeal carcinoma. BMC Med Genomics.

[16] Wuchty S, Arjona D, Bozdag S, Bauer PO (2012). Involvement of microRNA families in cancer. Nucleic Acids Res.

[17] Sengupta S, den Boon JA, Chen IH, Newton MA, Stanhope SA, Cheng YJ (2008). MicroRNA 29c is down-regulated in nasopharyngeal carcinomas, up-regulating mRNAs encoding extracellular matrix proteins. Proc Natl Acad Sci U S A.

[18] Xia H, Ng SS, Jiang S, Cheung WK, Sze J, Bian XW (2010). miR-200a-mediated downregulation of ZEB2 and CTNNB1 differentially inhibits nasopharyngeal carcinoma cell growth, migration and invasion. Biochem Biophys Res Commun.

[19] Deng M, Tang H, Zhou Y, Zhou M, Xiong W, Zheng Y (2011). miR-216b suppresses tumor growth and invasion by targeting KRAS in nasopharyngeal carcinoma. J Cell Sci.

[20] Alajez NM, Lenarduzzi M, Ito E, Hui AB, Shi W, Bruce J (2011). MiR-218 suppresses nasopharyngeal cancer progression through downregulation of survivin and the SLIT2-ROBO1 pathway. Cancer Res.

[21] Lu J, He ML, Wang L, Chen Y, Liu X, Dong Q (2011). MiR-26a inhibits cell growth and tumorigenesis of nasopharyngeal carcinoma through repression of EZH2. Cancer Res.

[22] Yi C, Wang Q, Wang L, Huang Y, Li L, Liu L, Zhou X, Xie G, Kang T, Wang H et al. MiR-663, a microRNA targeting p21(WAF1/CIP1), promotes the proliferation and tumorigenesis of nasopharyngeal carcinoma. Oncogene. 2012;31(41):4421–33. 10.1038/onc.2011.629 22249270

[23] Zeng X, Xiang J, Wu M, Xiong W, Tang H, Deng M (2012). Circulating miR-17, miR-20a, miR-29c, and miR-223 combined as non-invasive biomarkers in nasopharyngeal carcinoma. PLoS One.

[24] Wong QW, Lung RW, Law PT, Lai PB, Chan KY, To KF (2008). MicroRNA-223 is commonly repressed in hepatocellular carcinoma and potentiates expression of Stathmin1. Gastroenterology.

[25] Sun W, Shen W, Yang S, Hu F, Li H, Zhu TH (2010). miR-223 and miR-142 attenuate hematopoietic cell proliferation, and miR-223 positively regulates miR-142 through LMO2 isoforms and CEBP-beta. Cell Res.

[26] Li J, Guo Y, Liang X, Sun M, Wang G, De W, Wu W: MicroRNA-223 functions as an oncogene in human gastric cancer by targeting FBXW7/hCdc4. J Cancer Res Clin Oncol. 2012;138(5):763–74. 10.1007/s00432-012-1154-x PMC11824240 22270966

[27] Eto K, Iwatsuki M, Watanabe M, Ishimoto T, Ida S, Imamura Y (2015). The sensitivity of gastric cancer to trastuzumab is regulated by the miR-223/FBXW7 pathway. Int J Cancer.

[28] Gong B, Hu H, Chen J, Cao S, Yu J, Xue J (2013). Caprin-1 is a novel microRNA-223 target for regulating the proliferation and invasion of human breast cancer cells. Biomed Pharmacother.

[29] Nian W, Ao X, Wu Y, Huang Y, Shao J, Wang Y (2013). miR-223 functions as a potent tumor suppressor of the Lewis lung carcinoma cell line by targeting insulin-like growth factor-1 receptor and cyclin-dependent kinase 2. Oncol Lett.

[30] Streppel MM, Pai S, Campbell NR, Hu C, Yabuuchi S, Canto MI (2013). MicroRNA 223 is upregulated in the multistep progression of Barrett’s esophagus and modulates sensitivity to chemotherapy by targeting PARP1. Clin Cancer Res.

[31] Yang T, Zheng ZM, Li XN, Li ZF, Wang Y, Geng YF (2013). MiR-223 modulates multidrug resistance via downregulation of ABCB1 in hepatocellular carcinoma cells. Exp Biol Med (Maywood).

[32] Kasinski AL, Slack FJ (2011). Epigenetics and genetics. MicroRNAs en route to the clinic: progress in validating and targeting microRNAs for cancer therapy. Nat Rev Cancer.

[33] Lujambio A, Lowe SW (2012). The microcosmos of cancer. Nature.

[34] Zhou J, Yu L, Gao X, Hu J, Wang J, Dai Z (2011). Plasma microRNA panel to diagnose hepatitis B virus-related hepatocellular carcinoma. J Clin Oncol.

[35] Xu J, Yao Q, Hou Y, Xu M, Liu S, Yang L (2013). MiR-223/Ect2/p21 signaling regulates osteosarcoma cell cycle progression and proliferation. Biomed Pharmacother.

[36] Heegaard NH, Schetter AJ, Welsh JA, Yoneda M, Bowman ED, Harris CC (2012). Circulating micro-RNA expression profiles in early stage nonsmall cell lung cancer. Int J Cancer.

[37] Eychene A, Rocques N, Pouponnot C (2008). A new MAFia in cancer. Nat Rev Cancer.

[38] Vicente-Duenas C, Gonzalez-Herrero I, Garcia Cenador MB, Garcia Criado FJ, Sanchez-Garcia I (2012). Loss of p53 exacerbates multiple myeloma phenotype by facilitating the reprogramming of hematopoietic stem/progenitor cells to malignant plasma cells by MafB. Cell Cycle.

[39] Stralen E, Leguit RJ, Begthel H, Michaux L, Buijs A, Lemmens H (2009). MafB oncoprotein detected by immunohistochemistry as a highly sensitive and specific marker for the prognostic unfavorable t(14;20) (q32;q12) in multiple myeloma patients. Leukemia.

